# Assessment of exposure to influenza A viruses in pigs between weaning and market age

**DOI:** 10.1186/s13567-021-00927-9

**Published:** 2021-04-21

**Authors:** Juliana Bonin Ferreira, Zvonimir Poljak, Robert Friendship, Éva Nagy, Greg Wideman, Helena Grgić

**Affiliations:** 1 Department of Population Health and Pathobiology, North Carolina State University, Raleigh, NC USA; 2 Department of Population Medicine, University of Guelph, Guelph, ON Canada; 3 Department of Pathobiology, University of Guelph, Guelph, ON Canada; 4 South-West Veterinary Ontario Services, Stratford, ON Canada

**Keywords:** Influenza A virus, Antibodies, Swine, Co-circulation

## Abstract

Influenza A viruses (IAVs) are common causes of respiratory infection in pigs. The objective of this study was to characterize the circulation of IAVs between weaning and market age on the basis of development of antibody response and molecular epidemiology of detected viruses. Two batches of weaned pigs were followed in the nursery and finisher barns with a sample of 81 and 75 pigs. Nasal swabs and blood samples were collected from individual pigs for virological and serological analyses. A H3N2 subtype virus, of cluster IV, was detected in Study 1, with a maximum of 97.9% identity to HA gene of viruses previously isolated in Ontario. In Study 2, a H1N1 subtype virus, of 2009 H1N1 pandemic lineage, was detected, with a maximum of 97.8% identity to HA gene of viruses previously isolated in Ontario. On the basis of HA gene, it was observed that pigs were being detected with the same virus over time. The existence of antibody titers for IAV other than the isolated one confirmed that more than one subtype can circulate in the same population. In Study 1, pigs with higher numbers of IAV detection had lower serological titers for the same virus that was confirmed to circulate in the nursery (*P* < 0.01). Thorough knowledge of all endemic viral strains is fundamental for development of infection and disease control, particularly in complex production systems. This may include consideration of sampling and testing strategies which could detect circulation of all IAV variants, even if they have low prevalence.

**Supplementary Information:**

The online version contains supplementary material available at 10.1186/s13567-021-00927-9.

## Introduction

Influenza, an enzootic disease in many pig populations, is caused by influenza virus type A [1]. Three subtypes can be found circulating in pig barns worldwide: H1N1, H3N2 and H1N2. Prevalence of exposure at the herd level is generally very high. In previous studies, herd-level point prevalence of exposure has been estimated at 83% and 43% in Ontario sow and finisher herds, respectively [2]; and above 90% in sow herds in Belgium and Germany [3]. As in human influenza, the virus causes a self-limiting respiratory disease in individual animals. It has also been reported that the disease can spread quickly within the herd and disappear within 1–2 weeks [4]. In addition, different studies have reported that transport of weaned pigs can contribute to the dissemination of influenza A virus (IAV) among farms, and that sow herds and neonatal pigs might be important for the maintenance of the virus [5, 6].

Recent studies showed that swine influenza viruses can persist within a farm, causing two distinct outbreaks [7]. Additionally, recurrent infections with influenza A viruses in nursery pigs have been reported under field conditions in two different studies [1, 8]. Several studies have reported exposure of pigs to more than a single strain or subtype, either cumulatively or concurrently [1, 9, 10]. Simultaneous circulation of multiple distinct viruses can lead to reassortment.

Currently, the influenza situation is complex due to the existence of multiple strains that do not necessarily cross-react, as shown in multiple large scale studies [1, 11, 12]. However, even some within-herd studies suggest the presence of multiple strains [1, 9]. While studies focused on within-herd circulation of influenza virus under contemporary conditions continue to be limited, they are essential for understanding IAV ecology, and for designing infection control measures for IAV.

Therefore, the objective of this study was to characterize the circulation of IAV in pigs between weaning and market age on the basis of the development of antibody response and molecular epidemiology of detected viruses.

## Materials and methods

This research project was approved under University of Guelph AUP 3038.

### General overview

A detailed description of the barn included in this study has been previously provided [13]. Briefly, a nursery farm located in southern Ontario with a total capacity of 4000 animals was selected for the study. The farm was composed of two separate barns of equal capacity (2000 head each). Pigs from five sow-sources, belonging to the same multi-site swine production system, were weaned at approximately 19 days of age, and transported to this one nursery site.

### Study population

The nursery study barn selected for the two longitudinal studies had four equally-sized rooms with individual air flow, each room housing approximately 500 pigs in 24 pens, for a total barn capacity of approximately 2000 pigs. Two finisher sites, belonging to the same system, were included in the study. Finisher site for Study 1 comprised of one single barn, all-in-all-out, with capacity for 2000 pigs. Pens were all located in a single air-space with central hallway and were separated from the exterior by solid walls. Finisher site for Study 2 comprised of 2 barns, with the same characteristics as the one described in Study 1, but on a different geographical location. For the longitudinal studies, 81 piglets and 75 piglets were selected for Study 1 and Study 2, respectively [13]. In each study, the piglets originated from five different sow sources. For Study 1, blood samples were collected at entry to the nursery (3 week-old), and at the end of the nursery (10 week-old) from November 18^th^ 2013 to January 9^th^ 2014 and finisher periods (22–24 week-old) on April 1^st^, 11^th^ and 22^nd^, 2014. For Study 2, blood samples were collected at entry to the nursery (3 week-old), mid-nursery (6–7 week-old) and at the end of the nursery (10 week-old) from April 4^th^ 2014 to May 29^th^ 2014, and at the end of the finisher period (22–25 week-old) August 19^th^ and September 3^rd^ 2014. In addition to the pigs followed in Study 2, forty-one pigs not included in the longitudinal study were selected randomly in the finisher barn. The extra pigs were sampled for logistical reasons and because all the study pigs could not be located during the last sampling in the finisher barn.

### Serology

In order to determine the hemagglutination inhibition (HI) titers antibodies at entry to the nursery and also at the mid and end of the nursery period and end of finisher periods, serum samples were analyzed by the HI assay according to standard protocol [14] with four hemagglutinating units per well. The cut-off of HI titers was set at ≥ 1:40 as previously reported [14]. Six swine influenza isolates [15, 16] were used for HI: A/SW/ON/103–18/11/H3N2/HA, A/SW/ON/104–25/12/H3N2/HA, A/SW/ON/115–2/12/H3N2/HA, A/SW/ON/68/12/H1N2/HA, A/SW/ON/84/12/H1N1/HA, and A/SW/2/81/H1N1/HA, referred to throughout the article as H3N2_A, H3N2_B, H3N2_C, H1N2, H1N1_P, and H1N1_C, respectively. Also, two viruses identified in these longitudinal studies were used for HI: A/SW/ON/72–7-8/2014/H3N2 (Study 1) and A/SW/ON/148–9/2014/H1N1 (Study 2), referred to throughout the article as H3N2_H and H1N1_H, respectively. A complete description of the serological testing is reported elsewhere [13].

### Detection of influenza A virus

The presence of IAV in weekly nasal swabs as described in [13] was assessed by isolation and propagation of the virus in Madin-Darby canine kidney (MDCK) cells with added trypsin according to a standard protocol [17]. Virus replication was confirmed on the basis of the cytopathic effect (CPE) produced in the cells and also assessed by the hemagglutination assay according to a standard protocol [17]. Isolated viruses were then sequenced using the Illumina Miseq platform as described earlier [16] and subtyped on the basis of hemagglutinin and neuraminidase and sequenced using a deep sequencing approach. In selecting viruses for sequencing, priority was given to viruses that were detected in pigs with multiple infections over time. The consensus sequence was obtained after mapping the results of deep sequencing to the reference strains of H1N1 and H3N2 subtypes. The consensus sequence was used for further phylogenetic analysis. Phylogenetic trees were built using HA nucleotide sequences of included viruses. In addition, previous isolated Ontario strains and selected North American strains were included in the phylogenetic analysis. Sequence alignment was conducted using Clustal W algorithm with default parameters in Geneious R9.1. The similarity between isolates was calculated using the Jukes-Cantor approach, and an unrooted tree was built using the complete linkage approach.

### Statistical analysis

Descriptive statistics were generated for each variable and correlation was tested using the Spearman correlation coefficient. Titers were summarized through descriptive statistics and median and interquartile range (IQR) were presented. In addition, the difference in the median of the titers was compared for titers in the longitudinal study and in the cross sectional study (end of finisher), as described elsewhere [18].

Samples from Study 1 (three samplings) and Study 2 (four samplings) were then categorized in order to calculate incidence risk and prevalence determined by the presence of titers. For each of the eight viruses used in the HI assay, the binary results representing positivity at the start and the end of the nursery and at the end of the finisher phase were classified into four distinct groups: (0) pigs that did not have a positive titer in two subsequent measurements; (1) pigs that had a negative titer at the first measurement and a positive titer in subsequent measurements; (2) pigs that had a positive titer at the first measurement and a negative titer in subsequent measurements; (3) pigs that had a positive titer at the first and in the subsequent measurements. Proportion of pigs in each of the four latter groups was then described for each distinct period of measurement (e.g. between weaning and end of nursery period). In addition, incidence risk was calculated only if, in each of the distinct periods, at least 3 pigs tested negative for antibodies at the start of the period. Prevalence was calculated at the end of nursery and the end of finisher phase using number of animals with positive titers at the time of sampling as the numerator, and number of animals tested as a denominator. For the last measurement in Study 2, prevalence was determined for animals included in the longitudinal study, and additionally using animals that were sampled cross-sectionally.

Linear regression was performed to evaluate the association between the total number of virologically positive results during the nursery phase and the log_2_ HI antibody titers at the end of nursery period. The biological hypothesis evaluated here was that the intensity of infection, measured by the cumulative number of positive tests, influences the serological response at the end of the nursery period. Models were evaluated as described elsewhere [19].

Wilcoxon’s signed-rank test was performed to analyze the difference in serological response to each of the eight viruses between the measurements conducted at the end of the nursery and finisher phases. The biological hypothesis evaluated here was that time spent during the finisher phase would influence development of antibodies for the eight specific viruses in the finisher barn. Analysis was performed as described elsewhere [18]. Statistical analyses were conducted at the pig level using STATA IC 13 (StataCorp LP, College Station, TX, USA).

## Results

### Descriptive analysis

**Figure 1:**
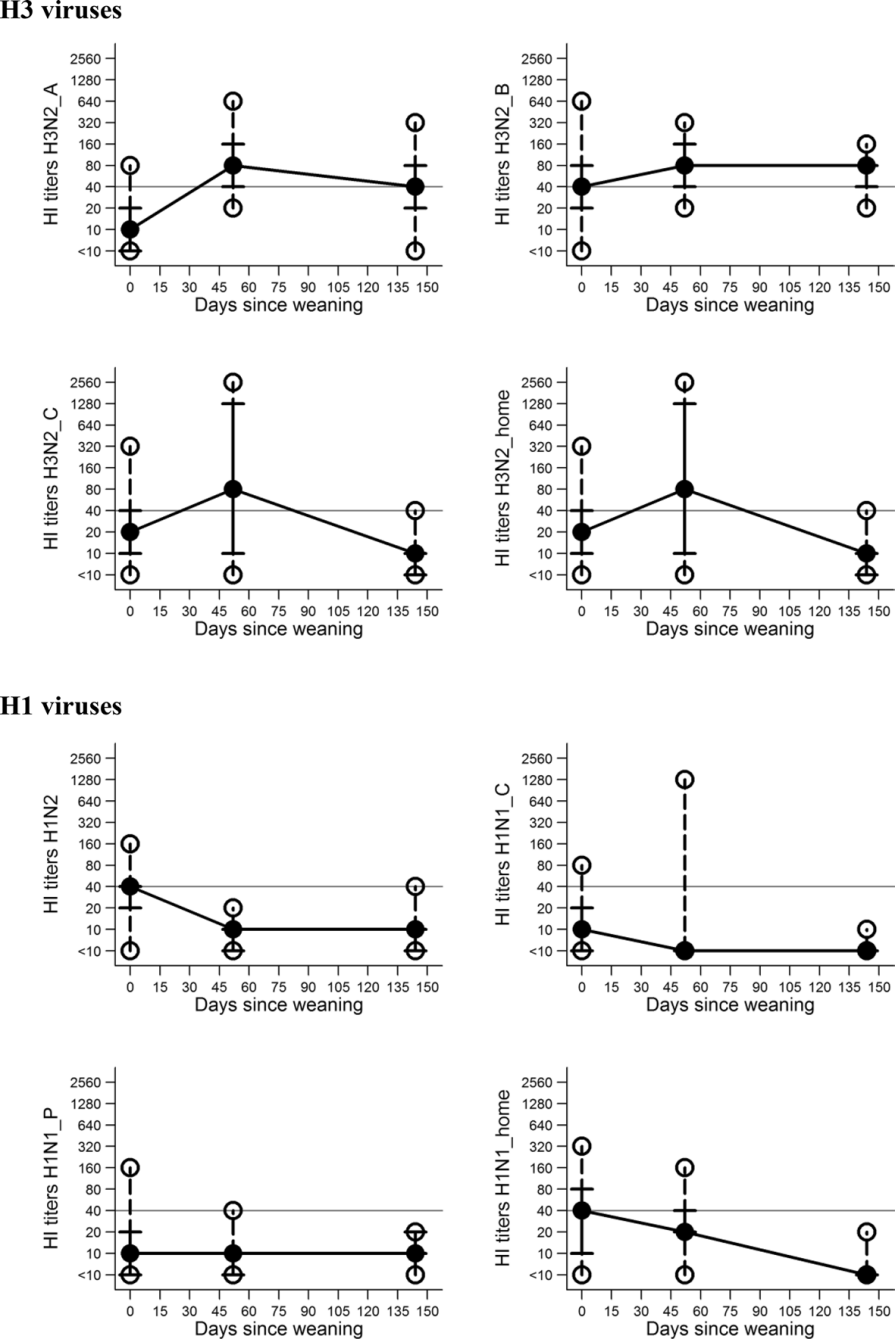
**Serological (HI test) titers of monitored pigs in three different measurements—Study 1.** Median represented by the solid black circle, p25 and p75 represented by the vertical lines, minimum and maximum represented by the hollow circles are shown representing the variability of titers throughout the study. The horizontal line represents the threshold for positive titers 1:40.

Descriptive analyses of all variables from both studies are presented in Table 1. In Study 1, eighty-one piglets were tested in the first measurement at entry to nursery, 79 in the second measurement at the end of nursery, and 57 in the third measurement at the end of finisher. In Study 2, seventy-five piglets were tested in the first measurement at entry to nursery, 74 in the second measurement in mid-nursery phase, 73 in the third measurement at end of nursery, and 39 in the fourth measurement at end of finisher. The fourth measurement included pigs in the longitudinal study (*n* = 39) and pigs selected randomly (cross-sectional *n* = 41) in the finisher barn. The median titers for the pigs included in the longitudinal study and those not included (cross-sectional) did not differ for most of the viruses tested, except H3N2_B and H1N1_H (Table 1). In both cases, titers of pigs randomly selected were higher than those of the pigs in the study. The results of serological testing of sera collected in all three measurements in Study 1 suggested variability in the antibody titers for all eight viruses tested (Figure 1 and Table 1). The titers for all H3 viruses increased during the nursery phase, while all H1 titers decreased or remained the same. Spearman correlation coefficients among the titers of different HI tests at each sampling occasion for study 1 are depicted in Additional file 1. At the entry to nursery, results showed a positive correlation amongst all titers with nine Spearman correlation coefficients ranging between 0.5 and 0.8. In the latter samplings correlation among titers varied in the direction and magnitude (Additional file 1).

**Table 1:** **Descriptive statistics of the measurements and frequency tables in the study of influenza A virus circulation**

Variable	Study 1		Study 2	
	Median (IQR)	N positive^±^ (%)	Median (IQR)	N positive (%)
H3N2_A^*^				
1^\\^	10 (15)	15 (18.5)	40 (40)	66 (88.0)
2	–	–	10 (10)	8 (10.8)
3	80 (120)	77 (97.4)	5 (0)	0 (0)
4 (pigs in study)	40 (60)	35 (61.4)	80 (0)	37 (97.3)
4^\^	–	–	80 (0)	78 (98.7)
H3N2_B^*^				
1	40 (60)	49 (60.4)	160 (80)	72 (96.0)
2	–	–	40 (20)	51 (68.9)
3	80 (120)	70 (88.6)	20 (10)	16 (21.9)
4 (pigs in study)	80 (40)	52 (91.2)	10 (10)	8 (21.0)
4	–	–	20 (70)	38 (48.1)
H3N2_C^*^				
1	20 (30)	39 (48.1)	80 (120)	61 (81.3)
2	–	–	20 (30)	20 (27.0)
3	80 (1270)	42 (54.4)	10 (5)	5 (6.8)
4 (pigs in study)	10 (5)	1 (1.7)	10 (5)	2 (2.5)
4	–	–	10 (5)	2 (5.2)
H3N2_H^†^				
1	40 (140)	60 (74.0)	80 (120)	67 (89.3)
2	–	–	20 (30)	19 (25.6)
3	80 (120)	63 (79.7)	10 (5)	3 (4.1)
4 (pigs in study)	20 (10)	12 (21.0)	5 (0)	1 (2.6)
4	–	–	5 (5)	3 (3.8)
H1N2^‡^				
1	40 (20)	43 (53.0)	80 (120)	62 (82.6)
2	–	–	20 (30)	22 (29.7)
3	10 (5)	0 (0)	5 (5)	4 (5.4)
4 (pigs in study)	10 (5)	1 (1.7)	10 (0)	1 (2.6)
4	–	–	10 (0)	3 (3.8)
H1N1_C^§^				
1	10 (15)	7 (8.6)	10 (15)	14 (18.6)
2	–	–	5 (0)	0 (0)
3	5 (0)	3 (3.8)	5 (0)	2 (2.7)
4 (pigs in study)	5 (0)	0 (0)	5 (5)	1 (2.6)
4	–	–	5 (5)	1 (1.2)
H1N1_P^¶^				
1	10 (15)	15 (18.5)	20 (30)	24 (32.0)
2	–	–	20 (10)	15 (20.2)
3	10 (5)	1 (1.2)	20 (30)	33 (45.2)
4 (pigs in study)	10 (10)	0 (0)	40 (40)	35 (92.1)
4	–	–	40 (40)	66 (83.5)
H1N1_H^†^				
1	40 (70)	42 (51.8)	20 (30)	22 (29.3)
2	–	–	5 (5)	4 (5.4)
3	20 (20)	39 (49.3)	80 (140)	54 (73.9)
4 (pigs in study)	5 (5)	0 (0)	20 (30)	15 (39.4)
4	–	–	40 (60)	45 (56.9)

The two longitudinal studies were performed in nursery pigs, between November 18^th^, 2013 and January 9^th^ and between April 4^th^, 2014 and May 29^th^, 2014, respectively. Fourth measurement was taken at the end of finisher period. Median and interquartile range were used. Any titer < 1:10 was treated, for the purpose of analysis, as 5.

^*^Different H3N2 variants broadly classified into cluster 4 of H3N2 swine influenza A virus and isolated in Ontario in 2012 and used in the hemagglutination inhibition assay.

^†^H3N2 and H1N1 viruses detected in the study and used as antigens in the hemagglutination inhibition assay.

^‡^H1N2 with hemagglutinin of the 2009 pandemic lineage and neuraminidase of the Cluster 4 H3N2 IAV-S.

^§^H1N1 IAV-S broadly classified as the classical swine H1N1 virus used in hemagglutinin inhibition assay.

^¶^H1N1 IAV-S of the 2009 H1N1 pandemic lineage used in the hemagglutination inhibition assay.

^\\^Number of blood sampling performed: (1) entry to nursery, (2) mid-nursery, (3) end of nursery, (4) end of finisher.

^\^Sum of pigs in the longitudinal study and pigs selected randomly in the finisher barn.

^±^Number of pigs that had a positive titer (≥ 1:40) in each of the measurements.

**Figure 2:**
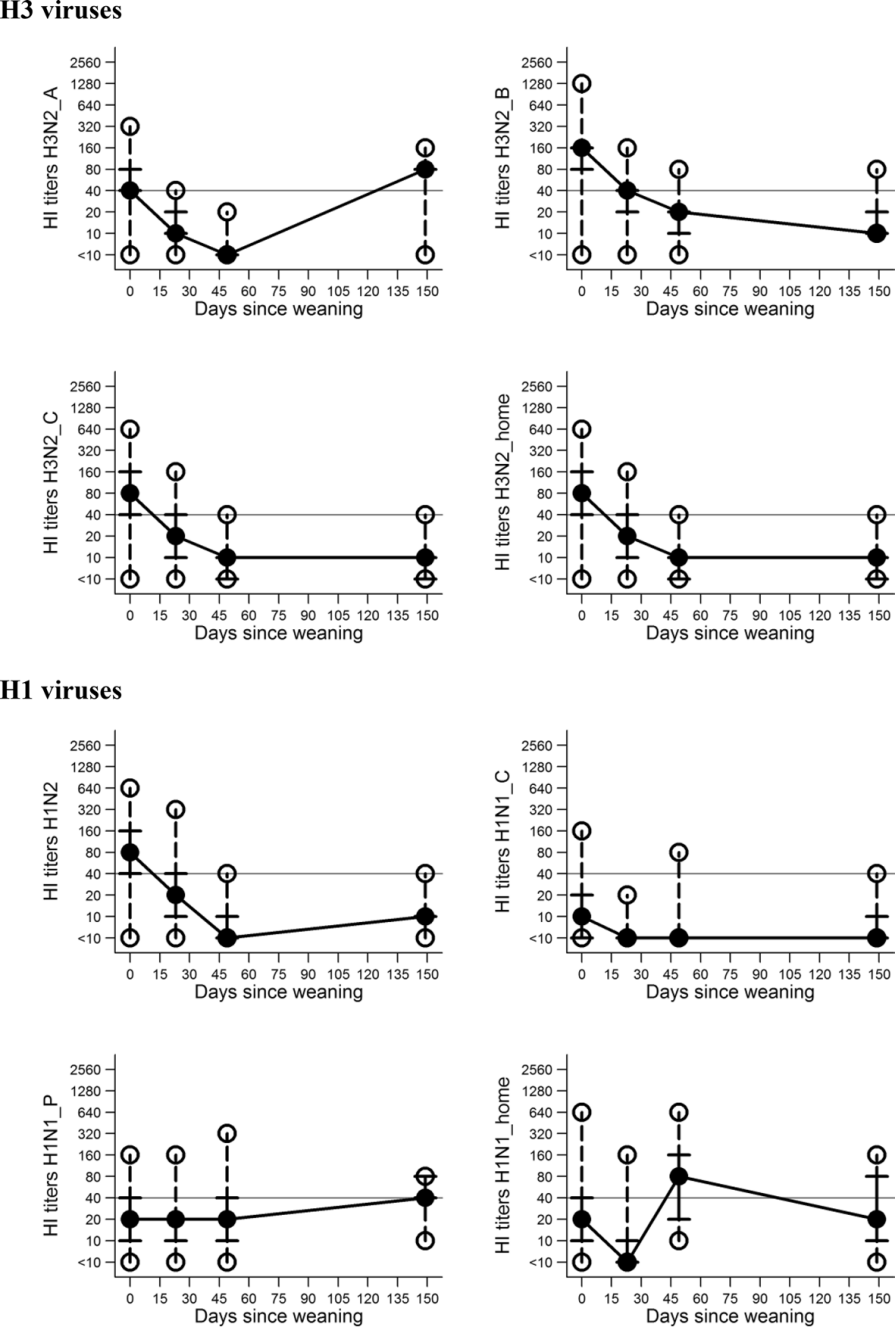
**Serological (HI test) titers of monitored pigs in four different measurements—Study 2.** Median represented by the solid black circle, p25 and p75 represented by the vertical lines, minimum and maximum represented by the hollow circles are shown representing the variability of titers throughout the study. The horizontal line represents the threshold for positive titers 1:40.

Serological results from samples collected in all four measurements in Study 2 suggested variability in the antibody titers for all eight viruses tested (Figure 2 and Table 1). Titers for H3N2_A decreased in the nursery, but increased in the finisher barn. For all the other H3 viruses, titers decreased or remained the same in both production sites. Titers for H1N1_P increased at the end of nursery and finisher phases, while H1N1_H titers increased at the end of nursery phase and decreased at the end of finisher phase. Spearman correlation coefficients among the titers of different HI tests at each sampling occasion for study 2 are depicted in Additional file 2. At the entry to nursery, strong positive correlation was observed among tests with different H3N2 viruses and H1N2 virus, and separately among H1N1 viruses. Descriptively, correlation weakened in absolute value over the duration of the study (Additional file 2).

Grouping of individual pigs into different categories on the basis of the change in titers for each virus during the nursery and the finisher phase in Study 1 and Study 2 is provided in Tables 2 and 3, respectively. In Study 1, the incidence for H3N2_A, H3N2_B, and H3N2_H in the nursery was 96%, 93%, and 95%, respectively. Point prevalence was also high: 97%, 88% and 79%, respectively, for these same viruses. In the finisher barn, the prevalence for H3N2_A and H3N2_B was 61% and 91%, respectively. Different results can be observed in Study 2. The incidence for H3N2_B (66%), H1N1_P (52%), and H1N1_H (76%) was high in the nursery phase. In the finisher phase, the incidence for H3N2_A (97%), H1N1_P (95%), and H1N1_H (76%) was also high. The prevalence for H1N1_H (73%) was high in the nursery phase. In the finisher phase, the prevalence for H3N2_A (97%), H1N1_P (92%) and H1N1_H (73%) was also high. Overall mortality in the barn was 1.8% and 1.9% for Study 1 and 2, respectively.

**Table 2:** **Incidence and prevalence in the nursery and finisher phase in Study 1 and frequency table indicating percentage of pigs with a distinct change in the serological status at the end of each production phase**

Phase of production and categories		%							
		H3N2_A^*^	H3N2_B^*^	H3N2_C^*^	H3N2_H^**†**^	H1N2^**‡**^	H1N1_P^**¶**^	H1N1_C^**§**^	H1N1_H^**†**^
Nursery	Incidence^**^ (%) (95% CI)	96 (0.89, 0.99)	93 (0.78, 0.99)	55 (0.38, 0.70)	95 (0.76, 0.99)	0 –	0 –	4 (0.01, 0.11)	48 (0.32, 0.65)
	Prevalence (%) (95% CI)	97 (0.91, 0.99)	88 (0.79, 0.94)	53 (0.41, 0.64)	79 (0.69, 0.87)	0 –	1 (0.01, 0.06)	3 (0.01, 0.10)	49 (0.37, 0.60)
	Proportion of study animals in strata defined by the serological exposure status at the start and end of study period								
	N–N^1^	2.4	2.4	22.0	1.2	43.9	78.1	84.2	24.4
	N–P^2*&^	75.6	35.4	26.8	24.4	0.0	0.0	3.7	23.2
	P–N^3^	0.0	8.5	23.2	18.3	52.4	17.1	8.5	24.4
	P–P^4*^	18.3	50.0	24.4	52.4	0.0	1.2	0.0	24.4
Finisher	Incidence (%) (95% CI)	–	–	0 –	0 –	1 (0.01, 0.09)	0 –	0 –	0 –
	Prevalence (%) (95% CI)	61 (0.47, 0.74)	91 (0.80, 0.97)	1 (0.01, 0.09)	19 (0.10, 0.31)	1 (0.01, 0.09)	0 –	0 –	0 –
	Proportion of study animals in strata defined by the serological exposure status at the start and end of study period								
	N–N	0.0	0.0	32.9	15.9	67.1	67.1	65.9	35.4
	N–P	1.2	9.8	0.0	0.0	1.2	0.0	0.0	0.0
	P–N	26.8	6.1	34.2	37.8	0.0	1.2	2.4	32.9
	P–P	40.2	52.4	1.2	14.6	0 (0)	0.0	0.0	0.0

^*^Different H3N2 variants broadly classified into cluster 4 of H3N2 swine influenza A virus and isolated in Ontario in 2012 and used in the hemagglutination inhibition assay.

^†^H3N2 and H1N1 viruses detected in the study and used as antigens in the hemagglutination inhibition assay.

^‡^H1N2 with hemagglutinin of the 2009 pandemic lineage and neuraminidase of the Cluster 4 H3N2 IAV-S.

^§^H1N1 IAV-S broadly classified as the classical swine H1N1 virus used in the hemagglutination inhibition assay.

^¶^H1N1 IAV-S of the 2009 H1N1 pandemic lineage used in the hemagglutination inhibition assay.

^1^Negative titers in both measurements.

^2*&^Negative titers in the measurement at the end of nursery and positive titers at the end of finisher. Used to calculate prevalence ^*^ and incidence risk^&^.

^3^Positive titers in the measurement at the end of nursery and negative titers at the end of finisher.

^4*^Positive titers in the measurement at the end of nursery and positive titers at the end of finisher. Used to calculate prevalence.

^**^Incidence was only calculated if more than three pigs were positive.

Total number of pigs at the end of nursery and end of finisher periods were 79 and 56, respectively. Pigs that died during the trial were treated as missing values.

**Table 3:** **Incidence and prevalence in the nursery and finisher phase in Study 2 and frequency table indicating percentage of pigs with a distinct change in the serological status at the end of each production phase**

Phase of production and categories		%							
		H3N2_A^*^	H3N2_B^*^	H3N2_C^*^	H3N2_H^**†**^	H1N2^**‡**^	H1N1_P^**¶**^	H1N1_C^**§**^	H1N1_H^**†**^
Nursery	Incidence (%) (95% CI)	0 –	66 (0.09, 0.99)	0 –	0 –	0 –	52 (0.37, 0.66)	3 (0.01, 0.11)	76 (0.63, 0.87)
	Prevalence (%) (95% CI)	0 –	21 (0.13, 0.33)	6 (0.02, 0.15)	4 (0.01, 0.11)	5 (0.01, 0.13)	45 (0.33, 0.57)	2 (0.01, 0.09)	73 (0.62, 0.83)
	Proportion of study animals in strata defined by the serological exposure status at the start and end of study period								
	N–N^a^	12.0	1.3	18.6	10.6	17.3	32.0	77.3	16.0
	N–P^b¥&^	0.0	2.6	0.0	0.0	0.0	34.7	2.6	53.3
	P–N^c^	85.3	74.6	72.0	82.6	74.6	21.3	17.3	9.3
	P–P^d¥^	0.0	18.6	6.6	4.0	5.3	9.3	0.0	18.6
Finisher	Incidence (%) (95% CI)	97 (0.86, 0.99)	26 (0.12, 0.45)	5 (0.01, 0.19)	2 (0.01, 0.14)	2 (0.01, 0.14)	95 (0.78, 0.99)	3 (0.01, 0.11)	76 (0.63, 0.87)
	Prevalence (%) (95% CI)	97 (0.86, 0.99)	21 (0.09, 0.37)	5 (0.01, 0.17)	2 (0.01, 0.13)	2 (0.01, 0.13)	92 (0.78, 0.98)	2 (0.01, 0.09)	73 (0.62, 0.83)
	Proportion of study animals in strata defined by the serological exposure status at the start and end of study period								
	N–N	1.3	29.3	42.7	46.7	46.7	1.3	77.3	16.0
	N–P	49.3	10.7	2.7	1.3	1.3	29.3	2.6	53.3
	P–N	0.0	10.7	5.3	2.7	2.7	2.7	17.3	9.3
	P–P	0.0	0.0	0.0	0.0	0.0	17.3	0.0	18.6

^*^Different H3N2 variants broadly classified into cluster 4 of H3N2 swine influenza A virus and isolated in Ontario in 2012 and used in the hemagglutination inhibition assay.

^†^H3N2 and H1N1 viruses detected in the study and used as antigens in the hemagglutination inhibition assay.

^‡^H1N2 with hemagglutinin of the 2009 pandemic lineage and neuraminidase of the Cluster 4 H3N2 IAV-S.

^§^H1N1 IAV-S broadly classified as the classical swine H1N1 virus used in the hemagglutination inhibition assay.

^¶^H1N1 IAV-S of the 2009 H1N1 pandemic lineage used in the hemagglutination inhibition assay.

^a^Negative titers in both measurements.

^b*&^Negative titers in the measurement at the end of nursery and positive titers at the end of finisher. Used to calculate prevalence ^¥^ and incidence risk^&^.

^c^Positive titers in the measurement at the end of nursery and negative titers at the end of finisher.

^d*^Positive titers in the measurement at the end of nursery and positive titers at the end of finisher. Used to calculate prevalence ^¥^.

Total number of pigs in the end of nursery and end of finisher periods were 73 and 38, respectively. Pigs that died during the trial were treated as missing values.

### Detection of influenza A virus

In order to present results for the detection of IAV, there is the need to briefly describe results showed in [13].

#### Study 1

**Figure 3:**
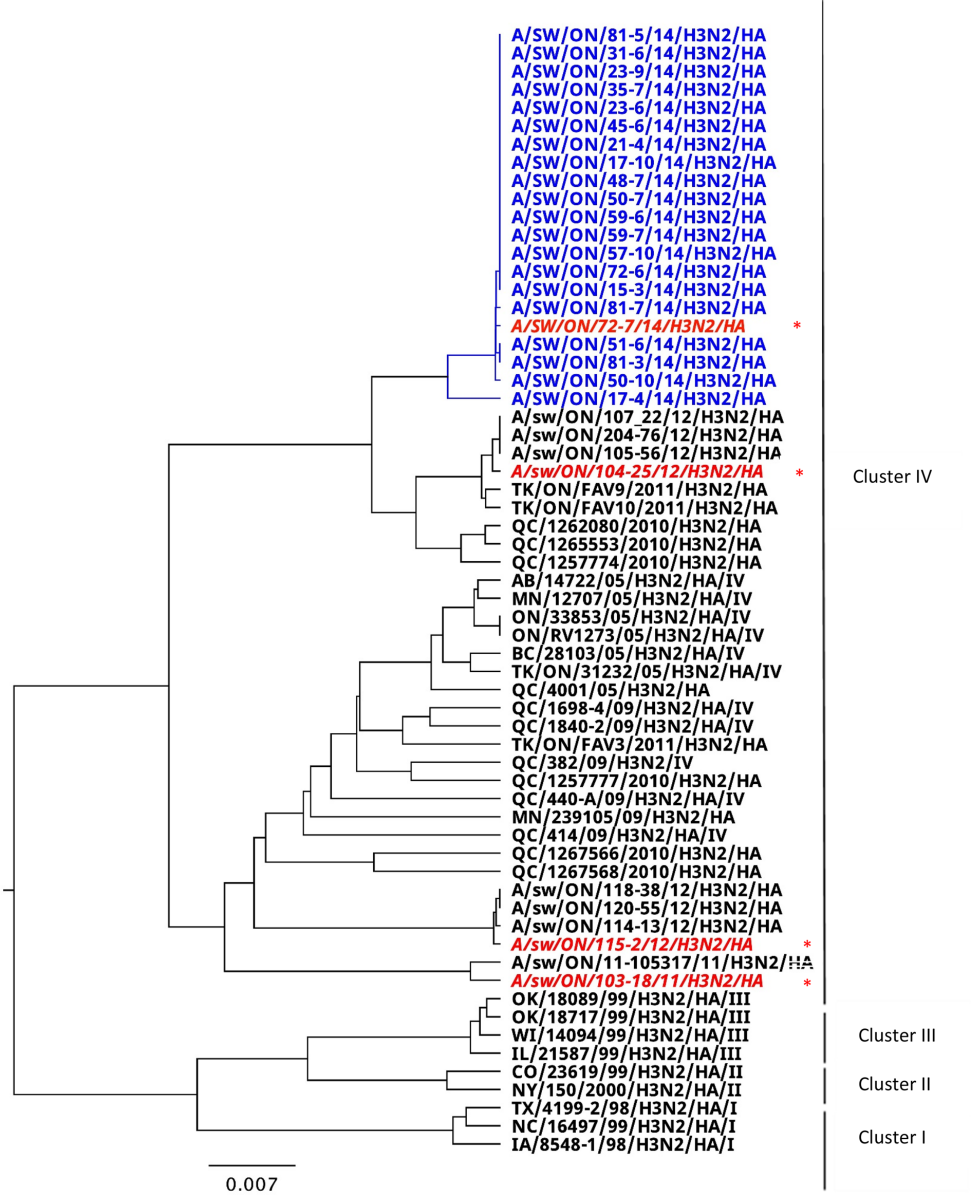
**Phylogenetic tree of the HA gene nucleotide sequences of H3N2 viruses in the longitudinal study during nursery phase.** The i**s**olated H3N2 viruses in Study 1 belong to cluster IV (in blue) (In descending orders starting with A/SW/ON/81-5/14/H3N2/HA and ending with A/SW/ON/17-4/14/H3N2/HA). Viruses used as reference strains in the hemagglutinin inhibition (HI) assay are labeled in red italicized font with asterisk, including one of the viruses isolated in the Study 1. Labels of the study viruses (e.g. A/SW/ON/81-5/14/H3N2/HA) contain unique pig identifier (e.g., 81 in the latter example), and sampling occasion (e.g., occasion 5 in the latter example). Pigs 17, 23, 50, 72, 81 had viruses sequenced on more than one occasion.

As previously shown, pigs were virologically positive up to four times. Pigs were sampled weekly during the nursery period, totaling 7 samplings (1 sampling/week), although sampling in the first week consisted of several sampling occasions to accommodate sampling of pigs shortly upon their arrival from different sow herds. Overall, of 81 pigs, 38 (46.9%) were positive once, 16 (19.7%) were positive twice, and 27 (33.3%) were positive three or more times [13]. Isolated viruses were sequenced and all isolates were characterized as identical or very similar viruses, herein named A/SW/ON/72–7-8/2014/H3N2. Phylogenetic analysis showed that the isolated viruses belonged to cluster IV on the basis of HA gene analysis (Figure 3). Identity of the majority of viruses was between 99.9 and 100% (i.e., maximum of 2 nucleotides), with only a single isolate having a minimum identity of 99.1% when compared to other viruses detected in Study 1 (Figure 3). However, when compared to other H3N2 viruses previously isolated in Ontario, identity was 93.8%, 97.9%, and 94.2% to H3N2_A, H3N2_B, and H3N2_C, respectively. Labels of the study viruses in Figure 3 (e.g. A/SW/ON/81–5/14/H3N2/HA) contain unique pig identifier (e.g., 81 in the latter example), and sampling occasion (e.g., occasion 5 in the latter example). Therefore, pigs that repeatedly tested positive for IAV and had their viruses sequenced, were found to have nucleotide sequences of the IAVs’ HA gene with 99.9% identity or higher, except in the case of one pig when the identity between viruses affecting the same pig on different occasions was 99.2% (Figure 3).

#### Study 2

**Figure 4:**
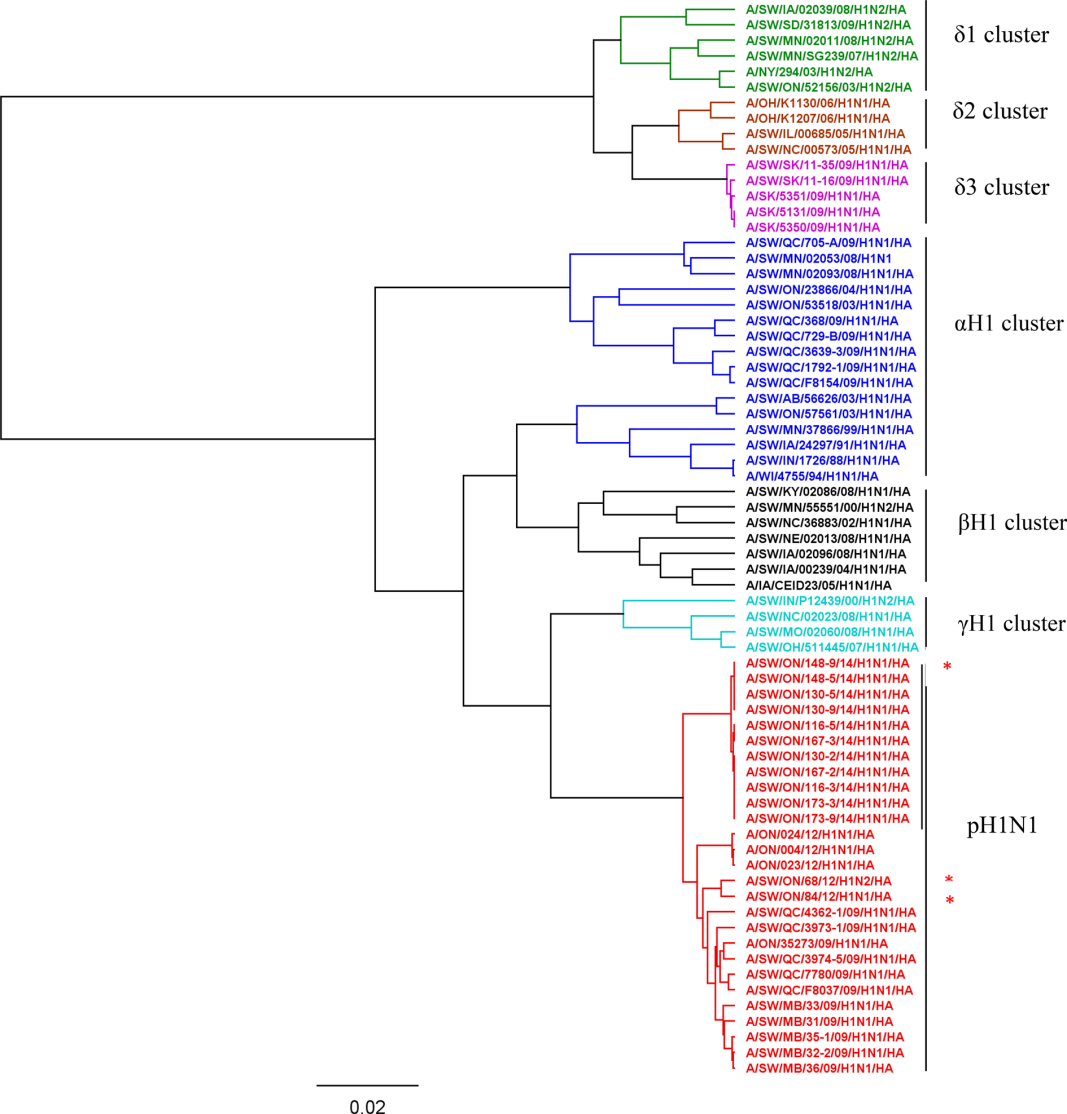
**Phylogenetic tree of the HA gene nucleotide sequences of H1N1 viruses in the longitudinal study during nursery phase**. The isolated H1N1 viruses in Study 2 are visualized in red color **(||)** and a part of pH1N1 cluster starting with A/SW/ON/148-9/14/H1N1/HA and ending with A/SW/ON/173-9/14/H1N1/HA. Viruses used as reference strains in the hemagglutinin inhibition (HI) assay are labeled with asterisk, including one of the viruses isolated in the Study 2. Labels of the study viruses (e.g. A/SW/ON/148-9/14/H1N1/HA) contain unique pig identifier (e.g., 148 in the latter example), and sampling occasion (e.g., occasion 9 in the latter example). Pigs 148,130, 173 had viruses sequenced on more than one occasion.

Overall, of the 75 pigs, virus was never identified from 27 (36%), whereas 36 (48%) were positive once, and 12 (16%) were positive two or more times. Isolated viruses were sequenced and all isolates were characterized as identical viruses, herein named A/SW/ON/148-9/2014/H1N1. Phylogenetic analysis showed that isolated viruses belong to cluster pH1N1 on the basis of HA gene analysis (Figure 4). Identity of the isolated viruses was a minimum of 99.82% with a maximum of three different nucleotides. However, when compared to other H1N1 previously isolated in Ontario identity was 97.8% and 97.7% for A/SW/ON/84/12/H1N1/HA, and A/SW/2/81/H1N1/HA, respectively. Labels of the study viruses in Figure 4 (e.g. A/SW/ON/148-9/14/H1N1/HA) contain unique pig identifier (e.g., 148 in the latter example), and sampling occasion (e.g., occasion 9 in the latter example).

##### Linear regression

**Figure 5:**
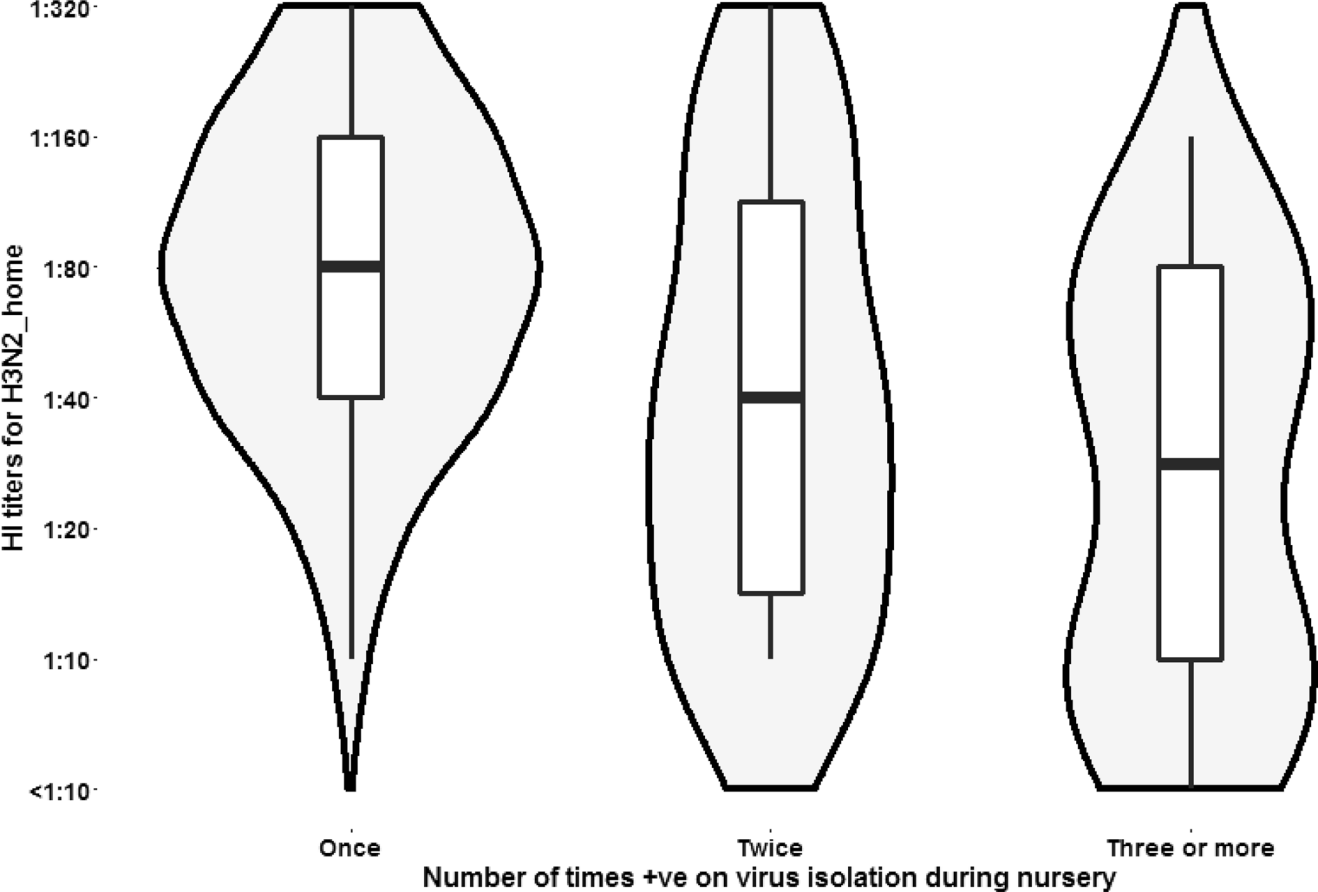
**Antibody response against H3N2_H among pigs positive with the virus at one, two, or three or more samplings.** The figure demonstrates that there is a negative dose–response relationship. The wider shape of the box/violin plot in the higher titer area of the figure is for pigs with one isolation of virus corresponds to the proportion of pigs with this result.

Table 4 shows results for the linear regression model for both studies. For Study 1, pigs with 3 or more positive results in the virological test had lower log_2_ titers than the referent category (*P* < 0.05) in the nursery barn for H3N2_H, also shown graphically in Figure 5. Similar result was observed for H3N2_B; while for H3N2_C a positive association between number of positive results and log_2_ H3N2_C titers was observed (*P* < 0.05). For all other variables, statistically significant results were not observed (data not shown). For Study 2, the opposite situation was observed for the same H3N2_H. Pigs with 2 or more positive results in the virological test had higher log_2_ titers than the referent category (*P* < 0.05). For all other variables, there was no statistically significant association between the number of positive results and log_2_ titers (data not shown).

**Table 4:** **Linear regression models for analysis of titers for influenza A based on the number of positive resu**lts

	Variable	Linear regression			
	Study 1	Coef	SE	*P*	95% CI
Nursery	H3N2_H^‡^			0.01^±^	
	2^\^	−0.9	0.44	0.03	−1.86, −0.08
	3^\\^	−1.6	0.37	0.01	−2.40, −0.92
	H3N2_C			0.01^±^	
	2	4.10	1.01	0.01	2.08, 6.13
	3	2.28	0.84	0.01	0.58, 3.97
	H3N2_B			0.03^±^	
	2	−0.54	0.32	0.10	−1.19, 0.11
	3	−0.68	0.27	0.01	−1.23, −0.14
	Study 2				
Nursery	H3N2_H^‡^			0.06^±^	
	1^¥^	0.06	0.22	0.78	−0.38, 0.50
	2^¥¥^	0.67	0.29	0.02	0.08, 1.27

^‡^The original titer divided by 10 and then log_2_ transformed.

^\^Pigs were positive twice. Reference category was that pigs were positive once.

^\\^Pigs were positive 3 or 4 times. Reference category was that pigs were positive once.

^¥^Pigs were positive once. Reference category was that pigs were never positive.

^¥¥^Pigs were positive twice or 3 times. Reference category was that pigs were never positive.

^±^*P*-value obtained by testing categorical variable using a partial likelihood test.

##### Wilcoxon’s signed-rank test

Results for the Wilcoxon’s signed-rank test are presented in Table 5. In Study 1, serological titers based on seven of eight viruses changed significantly over time. However, for six of these seven viruses statistically significant results were associated with the decrease in titers at the end of the finisher period. The only exception was H1N1_P, where an increase in titers for a larger number of pigs was observed (Table 5). In Study 2, serological titers based on five out of eight viruses changed significantly over time. In this study, the titers for H1N1_H predominantly decreased at the end of the finisher period (Table 5, *P* < 0.01; 66% of animals), whereas the titers for H3N2_A predominantly increased (Table 5, *P* < 0.01, 97% of animals). Such a result was consistent with the development of titers in pigs over a period of time as shown in Figure 2.

**Table 5:** **Wilcoxon paired signed-rank test of level of titers of pigs at the end of nursery period compared to level of titers at the end of the finisher period**

Variable	Study 1		Study 2	
	Observed	*P*-value	Observed	*P*-value
H3N2 A^*^				
Decreased titers^>^	35	0.01	1	0.01
Increased titers^>>^	8		37	
No difference^≠^	13		0	
H3N2_B^*^				
Decreased titers	32	0.01	15	0.76
Increased titers	14		12	
No difference	10		11	
H3N2_C^*^				
Decreased titers	39	0.01	11	0.57
Increased titers	7		14	
No difference	10		13	
H3N2_H^†^				
Decreased titers	51	0.01	15	0.02
Increased titers	2		5	
No difference	3		18	
H1N2^‡^				
Decreased titers	17	0.88	4	0.01
Increased titers	17		16	
No difference	22		18	
H1N1_P^¶^				
Decreased titers	11	0.05	10	0.01
Increased titers	24		25	
No difference	21		3	
H1N1_C^§^				
Decreased titers	15	0.01	3	0.07
Increased titers	2		9	
No difference	39		26	
H1N1_H^†^				
Decreased titers	54	0.01	25	0.01
Increased titers	0		1	
No difference	2		12	
Total^#^	56		38	

Comparison was made for the same pig having time as “treatment”.

^*^Different H3N2 variants broadly classified into cluster 4 of H3N2 swine influenza A virus and isolated in Ontario in 2012 and used in the hemagglutination inhibition assay.

^†^H3N2 and H1N1 viruses detected in the study and used as antigens in the hemagglutination inhibition assay.

^‡^H1N2 with hemagglutinin of the 2009 pandemic lineage and neuraminidase of the Cluster 4 H3N2 IAV-S.

^§^H1N1 IAV-S broadly classified as the classical swine H1N1 virus used in the hemagglutination inhibition assay.

^¶^H1N1 IAV-S of the 2009 H1N1 pandemic lineage used in the hemagglutination inhibition assay.

^#^Total number of the pigs sampled at the end of finisher.

^>^Pigs presented higher titers in the end of nursery when compared to the end of finisher.

^>>^Pigs presented lower titers at the end of nursery when compared to the end of finisher.

^≠^There is no difference in pigs titers at the end of nursery when compared to the end of finisher.

## Discussion

To expand our understanding of the development of antibodies to specific IAVs and what the factors might be to trigger the response, we combined repeated sampling and laboratory findings based on the combination of serological testing based on multiple influenza virus variants of the three common subtypes, including viruses detected in this specific barn.

This study confirmed that multiple influenza viruses were able to circulate in the same population during a relatively short period of time. All viruses in Study 1 were typed as H3N2 subtype and all were within a minimum of 99.7% identity of HA gene, although these viruses were obtained from the same animals that were repeatedly positive on virus isolation. This finding suggests that the same viable virus could be repeatedly detected from the same pig during a nursery phase over time period that extends typical period of infectiousness.

In Study 2, the H1N1 virus broadly classified into pandemic lineage A (H1N1)pdm09 emerged and was detected in pigs even on repeated samplings. This circulation of H1N1 virus might have been associated with management practices. Newly weaned pigs in Study 2 had relatively high HI titers for H3N2 viruses, likely because maternally derived antibodies (MDA) were derived through application of the H3N2-based autogenous vaccination of sows before farrowing. In this statement there is the assumption that the antibodies detected at entry to the nursery were due to MDA. The autogenous vaccine was implemented in response to repeated respiratory issues in the production system that was confirmed to be caused by different H3N2 IAV strains. Such vaccination strategy might have had an impact on decreasing the incidence of H3N2 infection, which could not be detected on the basis of virus isolation in Study 2. Nonetheless, H1N1 viruses started to circulate in the nursery and contribute to influenza incidence. In Study 1 and 2, circulation of different subtypes in the nursery was confirmed by genome sequencing of a number of viruses and by the increase in titers against H3N2 and H1N1 viruses at the end of nursery phase of Study 1 and Study 2, respectively.

Isolation of viruses from finisher pigs was not attempted for logistic reasons. However, development of antibody titers between the end of the nursery and the end of the finisher phase indicated further differences in influenza dynamics between the two cohorts. In the Study 1, titers for almost all viruses decreased, perhaps suggesting that there was no further significant circulation of any influenza viruses in the finisher barn. The H1N1_P was the only virus for which there was a statistical increase in titers between the end of nursery and the end of finisher phase; however, the prevalence of this virus based on formal declaration of a positive titer at the end of the finisher phase was 0% indicating that the statistical increase in titers was likely not of any practical significance. In Study 2, titers for the H1N1_H virus primarily decreased, suggesting that this virus did not circulate after the nursery phase while titers for the H1N1_P increased suggesting that virus of this lineage might also have been circulating in the finisher barn. In contrast, titers for H3N2_A increased in almost all pigs, suggesting that this virus was likely circulating in the finisher barn. This was the only H3N2 virus that showed such a marked increase, and was the virus that was only 93.8% identical to the H3N2_H virus isolated in Study 1. Taken collectively, these results therefore suggest that at least three distinct viruses were circulating in these 2 cohorts (H3N2_H, H1N1_H, and H3N2_A-like).

Circulation of multiple viruses in the same population is not a novel finding. A study conducted in Europe reported that co-circulation of different viruses is possible [1]. The authors reported that different subtypes were detected simultaneously in the same animal in all farms included in the study [1]. In another European study different H1N1 viruses were detected in the same farm during the study period [8]. Also in agreement with our findings, in a study conducted in Ontario antibodies for H3N2 and H1N1 viruses were detected on the same farms [10] and a recent American study also reports finding multiple viruses on the same farm [20]. However, the novelty of the present study was the in-depth investigation on the basis of different strains. The results imply that in the studied system, and possibly in other systems with similar complex animal flows, multiple viruses might circulate, creating complex patterns. Further research is needed to elucidate factors influencing such co-circulation, including the impact of cross-reactivity, maternal and active immunity, contact structure, and stochasticity. From the standpoint of practical infection control, the important issue is that the knowledge of all viruses and influenza virus genes in the production system need to be well understood before infection control measures are designed or implemented. Such knowledge should perhaps be gained by sampling and testing strategies that consider low prevalence of circulation for some influenza variants. Infection control measures based on the assumption of a single dominant virus might be oversimplification in complex systems. Another interesting finding was that the pigs which were positive by virus isolation on multiple occasions during the nursery phase had lower titers for the identical virus (H3N2_H) at the end of the nursery phase. Similar results were obtained for H3N2_B (97.9% identity with H3N2_H), but not for H3N2_C (94.2% identity with H3N2_H). This trend showed a negative dose–response relationship. This finding was interesting because it suggests that multiple infections with the same virus during the nursery phase do not necessarily lead to development of strong active immunity against that virus. In fact, multiple infections and low level of active immunity at the end of the nursery period might be a consequence of an inability of pigs to mount an effective immune response against a specific virus. This might be because of the presence of maternal immunity for a heterologous virus, which allows infection with a specific virus and prevents development of active immunity against that specific virus. Similar results were observed in an experimental study based on inoculation of sows with H1N1 and challenge of piglets with the same strain (strain-homologous). It has been observed [12] that pigs with passive immunity or MDA, coming from inoculated sows, expressed fewer and less severe clinical signs after inoculation with the same strain used to induce immunity in sows; however, they were not totally protected against infection. Moreover, active immunity was delayed or absent in the presence of MDA [12]. Another similar experimental study showed that the active immunity can be delayed when maternal antibodies are present [21]. It has been shown, in another study, that recurrent infections are possible in pigs with MDA for H1N1, H1N2 and H3N2 viruses when challenged with homologous IAV subtypes. It was also reported that pigs in the finisher barn seroconverted, showing that the absence of the MDA allowed active immunity to develop [1]. Researchers have also evaluated the ability of MDA to provide protection against a heterologous challenge and showed that the presence of MDA, for a specific virus, in non-vaccinated piglets, can suppress the development of active immunity (subtype-heterologous) [11]. In agreement with the findings of the present study, Allerson et al. suggested that IAV infection can occur in the presence of MDA. Groups of pigs were exposed to viruses that were subtype-homologous and subtype-heterologous to the MDA acquired from sow vaccination. Results showed that infection was present in pigs exposed to subtype-heterologous virus [22]. Another study showed that the presence of MDA did not prevent replication, however it did confer some clinical protection. MDA also impacted humoral and cellular responses, delaying the latter [23]. Findings from a different study showed that the presence of MDA can reduce IAV transmission, however, the reproduction number can be higher than 1, supporting that the presence of MDA might not protect against shedding of the virus [24]. All referenced studies showed that the presence of MDA can interfere with the humoral response and pigs were not fully protected against new infections.

### Limitations

Sequencing of all isolated viruses could not be performed due to high cost. In addition some assumptions could not be made for some of the analyses, such as the increase of titers when pigs were infected two or more times with H3N2_C in Study 1, and for H3N2_H in Study 2. Also, the viruses that were sequenced were isolated viruses and propagated in MDCK cells. Some viruses may not have replicated well in the culture or one virus out-competed others.

In conclusion, this study demonstrates that multiple strains and subtypes of influenza A viruses can circulate in the same pig population during a relatively short period of time. Factors that influence patterns of such circulation deserve to be studied in greater detail for a variety of reasons. From a practical infection-control perspective, thorough knowledge of all endemic viral strains and important influenza genes is needed as the basis for development of infection and disease control, particularly in complex production systems that seem to dominate in current swine production. This may include consideration of sampling and testing strategies which could detect circulation of all IAV variants, even if they have low prevalence.

## Supplementary Information

**Additional file 1. Spearman correlation coefficient of hemagglutination titers of pig sera tested by eight different antigens in a longitudinal Study 1 of influenza circulation in growing pigs. **Panel 1 represents entry to nursery, Panel 2 represents end of nursery phase, and Panel 3 represents correlation at the end of finisher phase.

**Additional file 2. Spearman correlation coefficient of hemagglutination titers of pig sera tested by eight different in a longitudinal Study 2 of influenza circulation in growing pigs.** Panel 1 represents entry to nursery, Panel 2 represents mid-nursery phase, Panel 3 represents end of nursery phase, and Panel 4 represents correlation at the end of finisher phase.

## Data Availability

Not Applicable—pork producers confidential data.

## Abbreviations

IAVs: Influenza A viruses
IAV: Influenza A virus
HI: Hemagglutination inhibition
MDCK: Madin-Darby canine kidney
CPE: Cytopathic effect
IQR: Interquartile range
MDA: Maternally derived antibodies
