# Supplementary figures and images for: Assessment of exposure to influenza A viruses in pigs between weaning and market age
Source: Vet Res. 2021 Apr 21;52:60. doi: 10.1186/s13567-021-00927-9 (PMC8059009; doi:10.1186/s13567-021-00927-9)

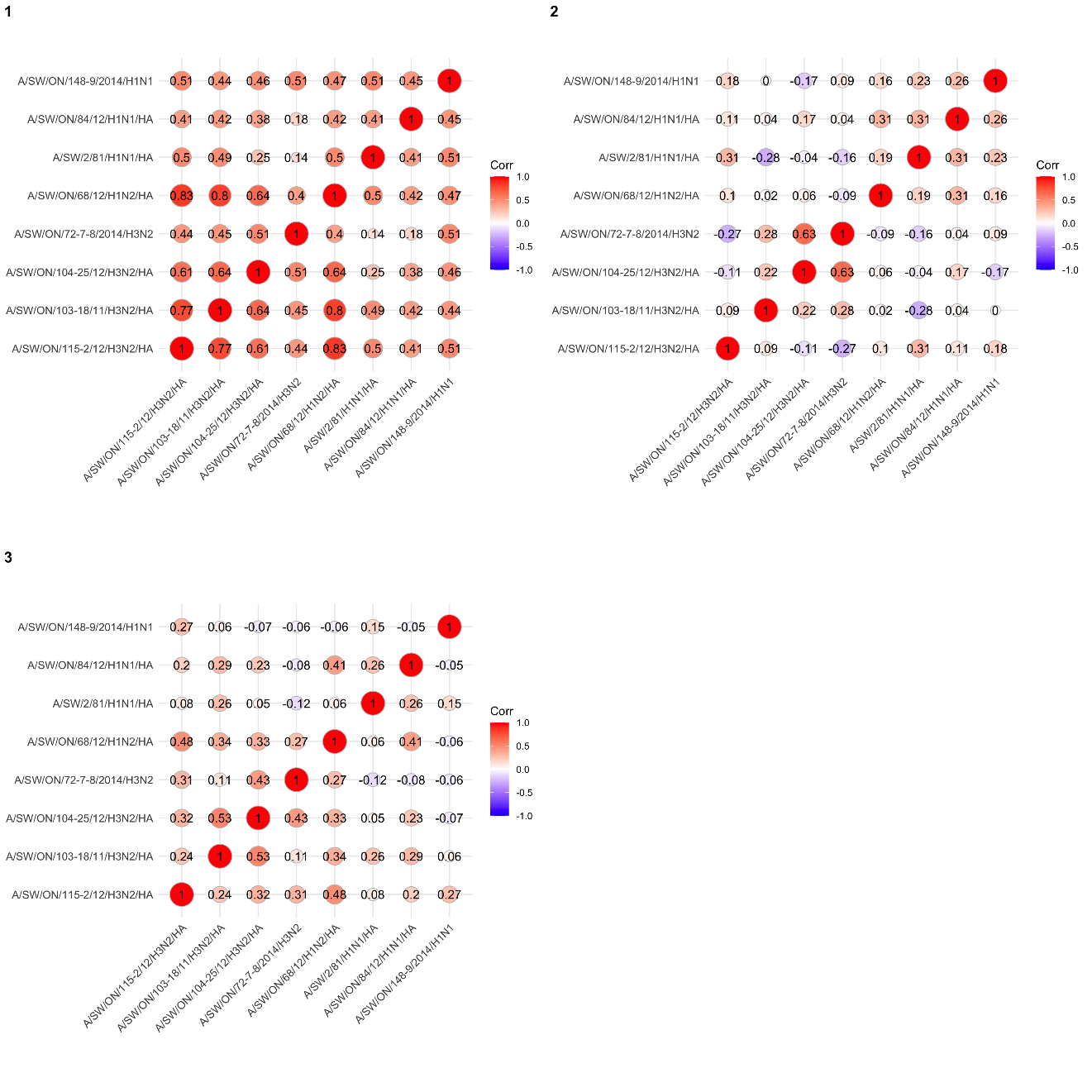

Supplement: Supplementary file 1 — Additional file 1. Spearman correlation coefficient of hemagglutination titers of pig sera tested by eight different antigens in a longitudinal Study 1 of influenza circulation in growing pigs. Panel 1 represents entry to nursery, Panel 2 represents end of nursery phase, and Panel 3 represents correlation at the end of finisher phase. [file 13567_2021_927_MOESM1_ESM.docx]

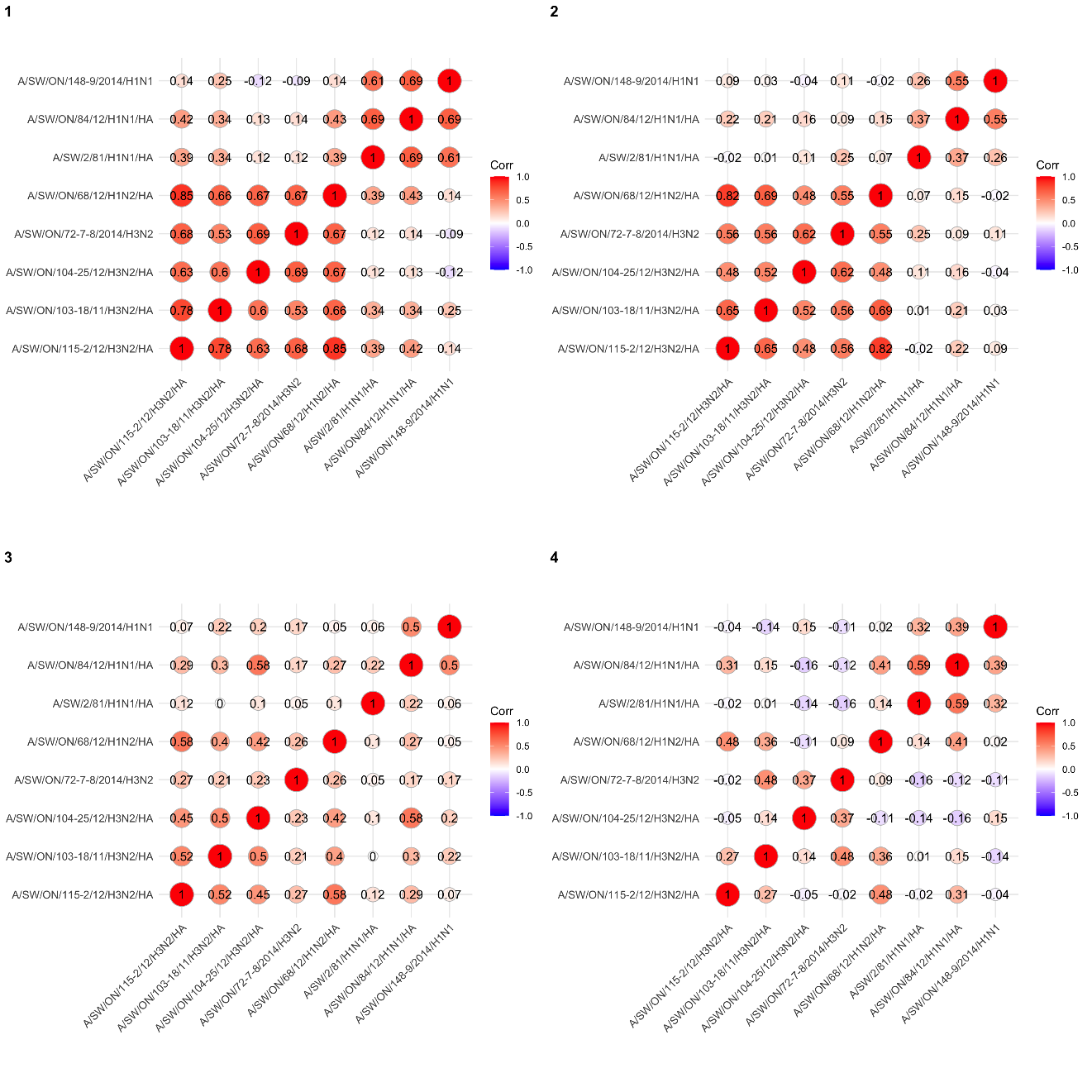

Supplement: Supplementary file 2 — Additional file 2. Spearman correlation coefficient of hemagglutination titers of pig sera tested by eight different in a longitudinal Study 2 of influenza circulation in growing pigs. Panel 1 represents entry to nursery, Panel 2 represents mid-nursery phase, Panel 3 represents end of nursery phase, and Panel 4 represents correlation at the end of finisher phase. [file 13567_2021_927_MOESM2_ESM.docx]
